# Natural Products for the Treatment of Post-stroke Depression

**DOI:** 10.3389/fphar.2022.918531

**Published:** 2022-05-30

**Authors:** Chaoyou Fang, Zeyu Zhang, Houshi Xu, Yibo Liu, Xiaoyu Wang, Ling Yuan, Yuanzhi Xu, Zhengyang Zhu, Anke Zhang, Anwen Shao, Meiqing Lou

**Affiliations:** ^1^ Department of Neurosurgery, Shanghai General Hospital, Shanghai Jiao Tong University School of Medicine, Shanghai, China; ^2^ Department of Neurosurgery, Second Affiliated Hospital, School of Medicine, Zhejiang University, Zhejiang, China; ^3^ Clinical Research Center for Neurological Diseases of Zhejiang Province, Hangzhou, China; ^4^ Department of Neurosurgery, Huashan Hospital, Shanghai Medical College, Fudan University, Shanghai, China

**Keywords:** natural products, post-stroke depression, treatment, HPA, hypothalamic-pituitary-adrenal, inflammation

## Abstract

Post-stroke depression (PSD) is the most frequent and important neuropsychiatric consequence of stroke. It is strongly associated with exacerbated deterioration of functional recovery, physical and cognitive recoveries, and quality of life. However, its mechanism is remarkably complicated, including the neurotransmitters hypothesis (which consists of a monoaminergic hypothesis and glutamate-mediated excitotoxicity hypothesis), inflammation hypothesis, dysfunction of the hypothalamic-pituitary-adrenal (HPA) axis, and neurotrophic hypothesis and neuroplasticity. So far, the underlying pathogenesis of PSD has not been clearly defined yet. At present, selective serotonin reuptake inhibitors (SSRIs) have been used as the first-line drugs to treat patients with PSD. Additionally, more than SSRIs, a majority of the current antidepressants complied with multiple side effects, which limits their clinical application. Currently, a wide variety of studies revealed the therapeutic potential of natural products in the management of several diseases, especially PSD, with minor side effects. Accordingly, in our present review, we aim to summarize the therapeutic targets of these compounds and their potential role in-clinic therapy for patients with PSD.

## 1 Introduction

Stroke is the leading cause of death and disability worldwide (Donnan et al., 2008). According to the World Health Organization, approximately 15 million people suffer a stroke annually (Mackay et al., 2004). As a major contributor to long-term disability in adults, stroke results in a need for long-term rehabilitation care and imposes an economic burden (Lin et al., 2014; Aoki et al., 2019; Wang et al., 2020a; Katzan et al., 2021). In addition, stroke always brings great psychological stress, which makes stroke patients vulnerable to depression.

Among the many neuropsychiatric effects of stroke, post-stroke depression (PSD) is the most prevalent and serious one. Over half of stroke survivors suffer from depression at some point (Ayerbe et al., 2013). Depression is strongly associated with compromised functional, physical, and cognitive recovery as well as poor quality of life (Li et al., 2017). The main clinical symptoms of PSD include depressed mood, apathy, weight loss or gain, sleep changes, a sense of worthlessness, anhedonia, and fatigue, the first two of which are the core symptoms (Feng et al., 2014). PSD is linked to an increased risk of short-term and long-term mortality (Bartoli et al., 2013). Moreover, clinical management of depressive symptoms has been demonstrated to be related to better functional recovery and more favorable outcomes (Chemerinski et al., 2001).

To date, the underlying pathophysiological mechanisms of PSD have not been elucidated (Lin et al., 2020). Numerous studies have suggested that the possible mechanisms include a change in ascending monoamine pathways, excess proinflammatory cytokines, dysfunction of the hypothalamic-pituitary-adrenal (HPA) axis, and alterations in neuroplasticity (Villa et al., 2018). Based on the classic monoamine hypothesis, selective serotonin reuptake inhibitors (SSRIs), serotonin and noradrenaline reuptake inhibitors (SNRIs), and some other antidepressants have been extensively developed and studied for the treatment of PSD (Villa et al., 2018; Effects of fluoxetine on functional, 2019). However, these antidepressants have various side effects, such as dizziness, sedation, and anticholinergic side effects (Sarko, 2000; Choi-Kwon et al., 2006). Furthermore, there is some controversy regarding the effectiveness of antidepressants in improving quality of life, function, and cognitive outcomes in patients with neurological disorders (Price et al., 2011). To minimize the side effects and maximize the therapeutic outcomes, alternative treatment strategies are urgently needed.

Compounds inspired by natural products may provide highly promising new resources for the treatment of PSD. Medicinal plants contain a wide variety of phytochemicals recognized for their therapeutic potential in the treatment of several diseases, including PSD (Zeng et al., 2017; Zhang et al., 2021a). In recent years, various phytochemical compounds with strong anti-depressant activity have been reported to exist in various herbal medicines (Shah et al., 2021). More importantly, natural products are often seen as ecologically sound and readily available resources with few side effects (Joshi et al., 2020). The potential of natural products in the treatment of PSD is a matter that warrants attention.

In this review, we focus on the potential pathogenesis of PSD and treatments in the context of recent evidence regarding available therapeutic procedures for depression. Then, we summarize the active compounds in natural products for the potential treatment of PSD and explore the novel therapeutic targets of these compounds. Finally, the perspectives of future research are reviewed in the outlook.

The following search terms were used: post-stroke depression, depression, natural products, Traditional Chinese medicine, treatments. Once we found the target article, we continued our search in the similar articles section of Pubmed. We evaluated studies in English that investigated interventions for natural products in depression. The current literatures on treatments with natural products for PSD were summarized in this review.

## 2 Pathophysiological Mechanisms of PSD

**FIGURE 1:**
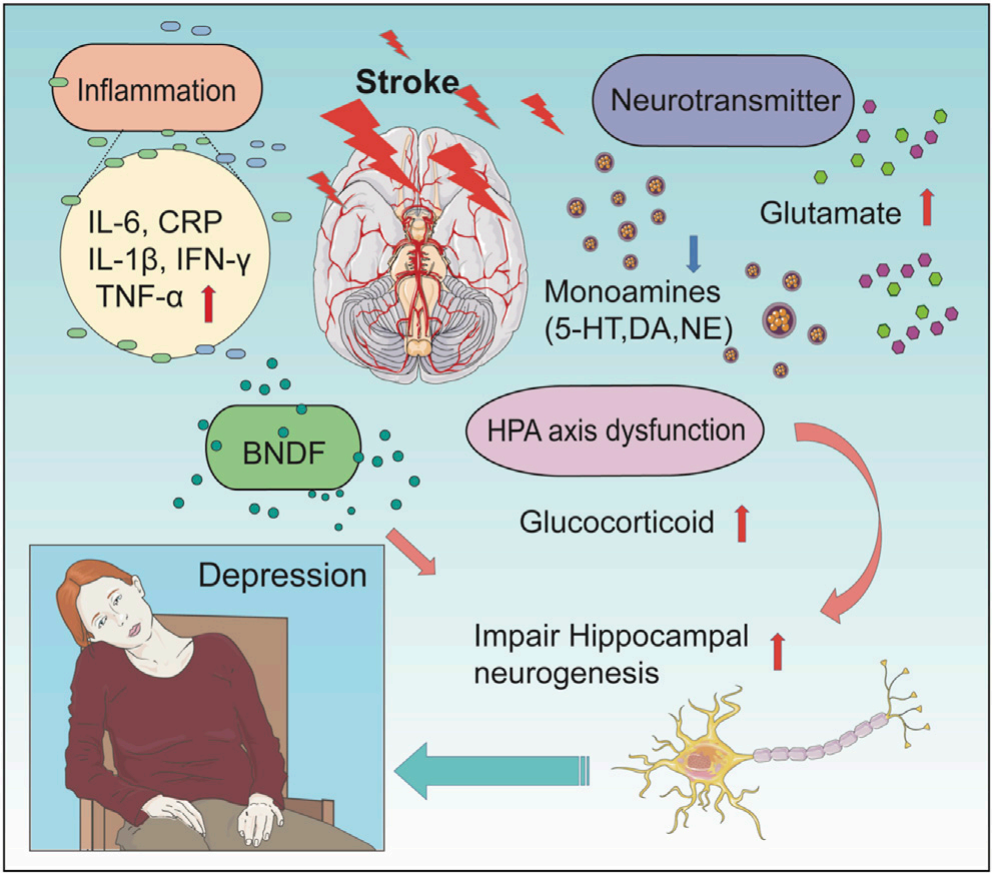
Pathophysiological mechanisms of PSD. The picture shows the association of the main pathophysiological mechanisms of PSD. There are four main mechanisms leading to PSD (including alterations in the neurotransmitter system, change in levels of inflammatory cytokine, dysfunction of the HPA axis, and influence of neuroplasticity). After a stroke, alterations of the neurotransmitter system include deduced level of monoamines (5-HT, DA, and NE) and an increased level of glutamate. Changes in levels of inflammatory cytokine include an increased level of IL-6, CRP, IL-1β, IFN-γ, and TNF-α. In PSD patients, the concentration of BNDF in the hippocampal was found to be decreased, which lead to impairment of hippocampal neurogenesis. At the same time, HPA axis dysfunction can lead to an increased concentration of glucocorticoid, which further impairs hippocampal neurogenesis. These changes can finally result in PSD.

The pathophysiology of PSD is remarkably complicated and, at present, poorly understood (Villa et al., 2018; Chen et al., 2020a); factors including both biological and psychosocial factors are likely to be involved. No single pathophysiological mechanism fully explains PSD (Loubinoux et al., 2012). Further research is needed to develop a better pathophysiological understanding of PSD to develop targeted interventions for prevention and treatment. Research supports the hypothesis that the disease process may be characterized by the reciprocal modulation of neurotransmitter systems, neuroinflammation, neuroendocrine activation, and neuroplasticity (Loubinoux et al., 2012). The main pathophysiological mechanisms of PSD are described in this section and illustrated in Figure 1.

Depressive behavior tests of forced swim test (FST) and tail suspension test (TST) were used in most of the research articles we included in the present review. FST and TST are commonly used to test for depression-like behavior, and screen for antidepressant properties in drugs, since antidepressants can cause immobile behavior to shorten (Porsolt et al., 1977; Steru et al., 1985; Toyoda, 2017). However, they are also usually used as animal models of depression (Gomez and Barros, 2000; Shinde et al., 2015). In fact, the majority of the studies that identify the antidepressants used TST and FST as animal models, including the articles we selected. In order to solve the problem that there are no animal models which both resemble depression and are selectively sensitive to clinically effective antidepressants, FST was reported as the first animal model for depression (Porsolt et al., 1977). TST as an animal model for screening antidepressants in mice was first recommended by Steru et al., in 1985 (Steru et al., 1985), which shares a common theoretical basis and behavioral measure with the FST. Although some researchers regarded FST and TST as only behavioral tests for screening antidepressants (AD) activity (Yan et al., 2010), some scholars hold that both of them are animal models of depression (Duman, 2010; Yi et al., 2011). According to previous research, we classify TST and FST as acute stress model (Toyoda, 2017). Besides FST and TST, some other animal models such as Chronic unpredictable mild stress (CUMS), Learned helplessness (LH) model, Olfactory bulbectomy (OBX) model, and Chronic social defeat stress (CSDS) model are also used in the articles we included.

### 2.1 Neurotransmitter Hypotheses

The neurotransmitter hypotheses have been regarded as the most significant set of mechanisms in PSD (Spalletta et al., 2006). At present, there are two leading concepts regarding the role of neurotransmitters in PSD: the monoaminergic hypothesis and glutamate-mediated excitotoxicity.

#### 2.1.1 Monoaminergic Hypothesis

For decades, studies have linked depression to monoamine levels (Moret and Briley, 2011). The monoamine hypothesis of depression is a classic model for understanding its pathogenesis (Tiemeier, 2003). In the 1960s, it was reported that monoamine deficiency in patients treated with reserpine for hypertension caused adverse reactions leading to depression, leading to the proposal of a neurochemical model of depression, which revealed that monoamine dysfunction in the central nervous system (CNS) has an association with depression (Schildkraut, 1965). Previous studies have firmly established an association between PSD and monoamine levels (Li et al., 2014). And monoamines are regarded as an important factor in PSD (Loubinoux et al., 2012; Espárrago Llorca et al., 2015). Monoamines, which mainly include 5-hydroxytryptamine (5-HT, or serotonin), dopamine (DA), and norepinephrine (NE), play a crucial role in the central nervous system (Chávez-Castillo et al., 2019). Previous studies have shown that 5-HT, NE, and DA neurons are interconnected in the CNS (Hamon and Blier, 2013). This phenomenon was first proposed by Robinson and Bloom in the 1970s, who also observed an association between decreased catecholamine concentrations and abnormal activity in rats (Robinson and Bloom, 1977). Additionally, a considerable number of studies have indicated that monoamine levels are lower in patients with depression than in those without depression. Some studies found that the brain 5-HT and NE levels of patients with depression were lower than those of patients without depression (Maurer-Spurej et al., 2007; Du et al., 2016). However, depressive symptoms can be effectively alleviated by increasing 5-HT bioavailability in synapses (Delgado et al., 1990).

Monoamines (5-HT, dopamine, and NE) are broadly distributed in the brain (Blier and de Montigny, 1994; Ji et al., 2014). Multiple antidepressant drugs (fluoxetine and tranylcypromine) can promote the release of dopamine and increase the expression of dopamine receptors to enhance dopaminergic function (Lammers et al., 2000). These increases in dopamine receptor expression are controlled by brain-derived neurotrophic factor (BDNF) (Guillin et al., 2001).

#### 2.1.2 Glutamate-Mediated Excitotoxicity Hypothesis

Glutamate-mediated excitotoxicity is another hypothesis of PSD. Although the role of glutamate concentrations in depression has been less extensively investigated than that of monoamines at both preclinical and clinical stages, there is still a great deal of research evidence on the role of glutamate in depression. There are studies indicating that glutamate is implicated in mood disorders (Veldic et al., 2019). Glutamate excitotoxicity is one of the main causes of neurodegeneration (Olivares-Bañuelos et al., 2019). Excitotoxicity caused by glutamate is reported to be important in several neurological and psychiatric disorders, including PSD (Mehta et al., 2013; Villa et al., 2018).

Several studies have shown that astrocyte cells can release gliotransmitters, including glutamate (Padmashri et al., 2015). In addition, astroglia expresses various glutamate receptors to respond to synaptically released glutamate (Morel et al., 2014). In stroke patients, high serum glutamate levels on admission are associated with stroke severity and poor prognosis. Additionally, high levels of glutamate after admission are independently associated with a worse disease course during the hospital stay (Hervella et al., 2020), which includes an impact on depression. Previous research has shown that alterations in glutamate recycling and glutamate receptors might be viable new possibilities for treating depression (Choudary et al., 2005), which provides potential ideas for the treatment of PSD patients.

### 2.2 The Inflammation Hypothesis

A growing body of convincing evidence shows that the inflammatory response, a vital biological event, has a strong relationship with depression (Ferrucci and Fabbri, 2018; Leonard, 2018). Increased inflammatory activation of the immune system is associated with stroke and depression, which negatively impacts health in both conditions (Wijeratne and Sales, 2021). The levels of multiple inflammatory factors in serum or plasma were frequently observed to increase in patients with PSD; examples include IL-6, CRP, IL-1, IFN-γ, IL-1β, and TNF-α (Yang et al., 2010; Su et al., 2012; Bensimon et al., 2014; Jiao et al., 2016; Kang et al., 2016). Thus, researchers suspected that inflammation was related to the occurrence of PSD and confirmed this hypothesis through animal experiments (Gibney et al., 2013).

Some researchers have even proposed the concept of depression as a dysfunction of the immune system (Leonard, 2010). The activated inflammatory response is hypothesized to be responsible for the high prevalence of depression following stroke (Pascoe et al., 2011). Cell death, including apoptosis and necrosis, might also be regulated by inflammatory cytokines (Gamdzyk et al., 2020). The growth of cerebral infarcts in vulnerable brain regions such as the hippocampus can be attributed to increased cell death in these areas. Depressive symptoms are also linked to increased cell death in these areas. (Kubera et al., 2011). As a result of cell death, large amounts of inflammatory cytokines are released (Rock and Kono, 2008). Consequently, inflammatory cytokines and cell death influence each other, further exacerbating the pathological process of depression.

This well-characterized cascade of inflammatory events occurs both in stroke and depression, which emphasizes the importance of proinflammatory cytokines (Adzic et al., 2018; Aleem and Tohid, 2018). The first prospective study on inflammatory cytokines and PSD has been conducted by Yang et al. (Yang et al., 2010). The authors found that an increased serum IL-18 concentration at the acute stage of stroke can be an independent risk factor for PSD. This was corroborated by reports that higher levels of TNF-α, IL-1B, IL-6, and IL-18 also independently predicted PSD occurrence in acute and subacute periods (Spalletta et al., 2006; Spalletta et al., 2013). A meta-analysis by Chen et al. showed that interleukin-6 (IL-6) and tumor necrosis factor-alpha (TNF-α) serum concentrations were higher in the PSD group than in the non-PSD group (Chen et al., 2020b).

There is evidence that antidepressants may reduce depressive symptoms by affecting immune markers and mood (Wijeratne and Sales, 2021). For instance, SSRI and SNRI administration was associated with increased levels of IL-10 (Ma et al., 2017). Similarly, an animal study demonstrated that IL-10 was increased after treatment with amitriptyline and fluoxetine (Qiu et al., 2017). In addition, the inflammation hypothesis is associated with the HPA axis. The HPA axis plays an important role in inflammation (Mahmood et al., 2020). HPA axis activation occurs in peripheral inflammation, leading to chronic stress-associated anxiety and depression (Sun et al., 2019). Several studies have shown that cytokines might induce hypercortisolism and glucocorticoid resistance through the inhibition of glucocorticoid receptors (Pace et al., 2011).

### 2.3 Dysfunction of the Hypothalamic-Pituitary-Adrenal (HPA) Axis

In psychiatry, abnormalities of the HPA axis have consistently been linked to depression, and the HPA axis is hyperactive in people who suffer from melancholic depression (Juruena et al., 2018). The HPA axis is a major component of the stress system that is involved in regulating mood (Glynn et al., 2013). When the HPA axis is activated, glucocorticoids are produced by the adrenal cortex (Li et al., 2016)*.* HPA axis dysregulation, leading to high cortisol reactivity, has been regarded as contributing to the somatic symptoms of depression (Chaplin et al., 2021).

Following a stroke, HPA axis activation, which is characterized by hypercortisolism, is quite common. (Johansson et al., 1997). Fassbander et al. found that acute ischemic stroke acts as a stressor and thus stimulates the HPA axis resulting in increased glucocorticoid levels (Fassbender et al., 1994). And studies have proved that serum cortisol levels were increased in PSD mice (Zhang et al., 2015a). Thus, we think that the Dysfunction of the HPA axis is one of the mechanisms of PSD. However, the specific mechanism of hypercortisolism-related depression is still unclear (Feng et al., 2014). There are insights that depression is associated with hypercortisolism and glucocorticoid receptor dysfunction (Carvalho and Pariante, 2008), while Weidenfeld et al. argue that the effect of glucocorticoids on stroke might improve neurological outcomes (Weidenfeld et al., 2011).

Moreover, the hippocampus is a key component linked to the HPA axis (Jacobson and Sapolsky, 1991). Corticosterone, the final product of the HPA axis, has a major target in the hippocampus, and the action of this hormone on the hippocampus is correlated with the pathogenesis and progression of depression (Saveanu and Nemeroff, 2012). According to the experiment of Zhang et al., we hypothesized that drugs used for inhibition of corticosterone-induced neurotoxicity in the hippocampus can be applied for clinical treatment of PSD (Zhang et al., 2021b).

### 2.4 Neurotrophic Hypothesis and Neuroplasticity

The neurogenesis hypothesis is relatively new when compared with other hypotheses about depression. According to the neurogenesis hypothesis, depression is largely the result of impaired nerve growth in the brain and the impairment of the brain’s ability to promote neurogenesis is the root cause of depression (Yan et al., 2016).

A number of correlative studies support the hypothesis that new neurons of the hippocampus play an essential role in mood control and the efficacy of antidepressants. Researchers have discovered that patients with depression suffer from lower neurogenesis and hippocampal volume, whereas antidepressants can increase neurogenesis in the hippocampus (Eisch and Petrik, 2012). It appears that hippocampal alterations play a critical role in the pathogenesis of depression, according to a variety of studies (Frodl et al., 2002). Hippocampal volume loss is a hallmark of clinical depression and restoration of adult hippocampal neurogenesis leads to recovery in depression patients (Sheline et al., 1996; Jacobs et al., 2000). The effects of chronic stress-induced depression on adult hippocampal neurogenesis have been demonstrated in rodent studies (Sahay and Hen, 2007). Additionally, rodents with impaired hippocampal neurogenesis leads to the development of depression-like behaviors, partly due to the fact that hippocampal neurogenesis buffers the over-reactivity of the HPA axis when stress is present (Snyder et al., 2011; Egeland et al., 2015).

Nevertheless, antidepressants and alternative antidepressant interventions stimulate adult hippocampal neurogenesis, which could play a role in treatment outcomes. Fang et al. presumed that potential therapeutic strategies for depression may include increased neurogenesis (Fang et al., 2020). Neurogenesis and synaptic plasticity in the hippocampus can be impaired by chronic excess glucocorticoids (Stranahan et al., 2008; Anacker et al., 2013). Reduced neurogenesis is linked to stress and depression, but antidepressant therapy exhibits anti-depressive effects and increases neurogenesis (Mahar et al., 2014). Depression is always accompanied by decreased hippocampal neurogenesis, while chronic antidepressant treatments (such as fluoxetine) can upregulate hippocampal neurogenesis (Wang et al., 2008a).

At the same time, some studies found that hippocampal volume decreased in stroke patients compared with healthy controls. Khlif et al. found that Atrophy rates for first-ever strokes, especially those with ipsi-lesional hippocampal atrophy, are much higher than for healthy controls and contra-lesional strokes (Khlif et al., 2019). Werden et al. found a smaller hippocampal volume in first-ever stroke patients than in healthy controls, and the hippocampal volume was smaller in patients with recurrent strokes than in controls and patients with their first stroke (Werden et al., 2017). These studies suggest that upregulating hippocampal neurogenesis might be a potential treatment for PSD patients.

Brain-derived neurotrophic factor (BDNF), as an endogenous neurotrophic factor, is necessary to modulate neuronal plasticity, inhibiting cell death cascades and increasing cell survival proteins. In the central nervous system, it maintains and promotes the proliferation of neurons (Benarroch, 2015). BDNF is an important regulator of synaptogenesis, neurogenesis, synaptic plasticity, and cell survival underlying memory and learning (Sairanen et al., 2005). The rate of hippocampal neurogenesis was increased by BDNF. Nevertheless, it has been observed that blood and hippocampal BDNF levels drop in patients with depression (Aydemir et al., 2006; Castrén et al., 2007). Antidepressant drug effects correlate with increased BDNF synthesis and activity in the hippocampus (Chen et al., 2001; Vásquez et al., 2014). Accordingly, BDNF has been proposed as one of the major candidate targets for the treatment of the antidepressant response.

In summary, the pathophysiology of PSD is complex and likely influenced by both biological and psychosocial factors. As previously described, the pathophysiology of PSD does not exist in isolation in the pathogenic process. For example, inflammation and the HPA axis influence each other. The HPA axis is one major regulator of inflammation, and a stress-induced release of cytokines, like IL-6, activates the HPA axis, which leads to rapid increases in adrenocorticotropic hormone (ACTH) and cortisol (Bethin et al., 2000; Mahmood et al., 2020). Dopamine receptors are strongly associated with the inflammasome signaling pathway (Wang et al., 2020b). Moreover, it was discovered that dopamine receptors are critical therapeutic targets in immunoregulation and inflammation (Taylor et al., 2003). Some monoamine reuptake inhibitors applied in clinical practice have been found to increase the expression of BDNF in depression-like rodent models (Molteni et al., 2006). A better understanding of PSD pathophysiology is essential to developing targeted prevention and treatment interventions.

## 3 Traditional Treatments for PSD

Although its precise mechanisms remain to be delineated, the treatment of PSD has made great progress. Traditional methods of treating PSD remain pharmacotherapy and psychological therapy. The most effective method of treating PSD is pharmacotherapy because of its high efficacy. In general, SSRIs are the first line of treatment for PSD, and tricyclic drugs are the second line of treatment (Castilla-Guerra et al., 2020).

Previous studies have indicated that antidepressants improve the recovery from the disability of patients treated with them than those not treated with antidepressants, and cognitive skills and functional recovery can improve with antidepressants in patients with PSD. Antidepressant treatment initiated early in non-depressed stroke patients can improve their cognitive and functional recovery and reduce their risk of developing post-stroke depression (Mead et al., 2015).

Several studies have examined the efficacy of pharmacotherapy or psychotherapy to prevent PSD. The introduction of TCAs and MAOIs in the 1950s revolutionized the treatment of depression (Pacher and Kecskemeti, 2004). With the development of newer agents, SSRIs have become the first-line drugs for the treatment of depression among several other indications (Dremencov et al., 2009). In animal models of stroke, SSRIs have shown convincing benefits (McCann et al., 2014). Among them, duloxetine (a serotonin and norepinephrine reuptake inhibitor) has been found to be an effective antidepressant when compared to a placebo and to be equally effective as various SSRIs (Girardi et al., 2009). Multiple clinical trials have been conducted since then to evaluate the effectiveness of antidepressants in PSD. The studies above indicated that all pharmacological treatments are SSRIs (e.g., escitalopram), SNRIs (e.g., duloxetine or venlafaxine), and tricyclic drugs (e.g., nortriptyline). Recently, a network meta-analysis that included all RCTs published between February 1984 and October 2016 assessed and ranked the efficacy and tolerability of antidepressants for PSD. The most acceptable treatments are paroxetine, placebo, sertraline, and nortriptyline based on the acceptability ranking (Deng et al., 2017). Nevertheless, a recent network meta-analysis, including 14 RCTs, examined the efficacy, acceptability, and tolerability of antidepressants in the treatment of PSD. The results showed that the major antidepressants did not display a significantly increased effectiveness when the efficacy of different antidepressants was compared (Qin et al., 2018). Some drugs, such as agomelatine, have a rapid antidepressant action and a high degree of safety and tolerability that probably enhances compliance with treatment. In brain areas such as the hippocampus and prefrontal cortex, agomelatine improves neuroplasticity mechanisms as well as adult neurogenesis (Pompili et al., 2013).

For the moment, there is still controversy about the best drug to treat PSD from those currently available. PSD treatment still leaves many questions unanswered, such as the most useful medications and the best timing for treatment. Additionally, the adverse effects of current pharmacotherapy cannot be ignored. Although antidepressants showed therapeutic effects in patients with PSD, we must also take into account other issues. For example, obvious complications often occur, such as addiction, toxicity, reduced effectiveness over time, relapse, and recurrence concerns (Sarko, 2000).

## 4 Potential and Mechanisms of Natural Products in the Treatment of PSD

Natural products are a potential source of drugs for nervous system-related disorders, including PSD (Zhang et al., 2021a). Being easily accessible and having few side effects, natural products are seen as having the potential for the therapeutic use of PSD (Joshi et al., 2020). Thus, it becomes imperative to search for novel therapies without side effects based on natural products. Even though drug design and discovery rely heavily on synthetic chemistry, the contribution of natural products cannot be ignored. In this part, we aim to identify the active compounds from natural products for the promising treatment of PSD by different types of mechanisms. The therapeutic effects of some natural products occur through a variety of mechanisms.

### 4.1 Therapeutic Strategy of Modulation in Neurotransmitters

As described previously, the neurotransmitter hypothesis has been regarded as the most significant mechanism in PSD, while monoamines are the most studied and mainly include 5-HT, DA, and NE. Anti-neurotransmitter therapy has been widely found in many natural products. Numerous studies have indicated that numerous natural products exert antidepressant effects by acting on neurotransmitters; however, most of those studies could not draw firm conclusions. Further studies are needed to reveal the exact antidepressant mechanism.

Monoamine levels were lower in patients with depression than in patients without depression, which is partly due to monoamine oxidase. Monoamine oxidase (MAO) degrades several monoamine neurotransmitters (Chen et al., 2011). When individuals suffer from depression, the level of monoamine oxidase enzyme in the brain is increased, which in turn reduces levels of monoamines and aggravates the symptoms of depression. Enzyme inhibition has already led to the discovery of a wide variety of useful natural products in the treatment of PSD. Several studies have found that some natural products produce antidepressant effects through the inhibition of MAO. All of the animal models used in this research involved one or both the FST and TST models. In a study by Zhu et al., they found that baicalin reduced immobility time in the FST and TST in mice. Baicalin, at doses of 12.5, 25, and 50 mg/kg (p.o.), also decreased immobility time in the FST in rats, which may act through MAO-A and MAO-B inhibition (Zhu et al., 2006). Kaempferol, apigenin, and chrysin proved to be potent monoamine oxidases (MAOIs); however, they produced more pronounced inhibition of MAO-A than MAO-B and exerted monoamine catabolism and neuroprotection on the rat brain. Sloley et al. found that kaempferol reduces the immobility time in the FST and TST when administered at a dose of 30 mg/kg (o.p.), possibly acting via MAO inhibition (Sloley et al., 2000). Similar studies have been abundant in the last few decades. For example, methanol extract of the roots of *Sophora* flavescens, curcumin, fisetin, trans-resveratrol, punarnavine, total glycosides of peony (TGP), isorhynchophylline (IRN), and cocoa polyphenolic extract have been found to inhibit MAO (Hwang et al., 2005; Mao et al., 2008a; Kulkarni et al., 2008; Messaoudi et al., 2008; Xu et al., 2010; Zhen et al., 2012; Dhingra and Bhankher, 2014; Xian et al., 2017).

Some studies have found that some natural products exert their antidepressant effects by upregulating the level of 5-HT or 5-HT receptors; some such natural products act entirely through a single mechanism. Depending on an interaction with the serotonergic 5-HT1A receptors, hesperidin decreased the immobility time in the FST and TST without affecting locomotor activity in the open-field test (OFT) (Souza et al., 2013). Neferine has also been found to have antidepressant activities by stimulating 5-HT1A receptors (Sugimoto et al., 2010). Through direct or indirect facilitation of central serotonergic transmission, extract of *Tagetes* lucida and the butanol and chloroform fractions from *Hypericum* canariense and *Hypericum* glandulosum exert antidepressant-like effects in mice (Sánchez-Mateo et al., 2005; Guadarrama-Cruz et al., 2008; Sánchez-Mateo et al., 2009). In addition, by stimulating neuronal 5-HT2A receptors, 1-(m-chlorophenyl) piperazine (1 mg/kg, i. p.) exhibited depressant-like effects in the FST and TST (in mice) without affecting locomotor status (Rajkumar et al., 2009).

Moreover, one study had an opposite finding, in which the downregulation of the 5-HT2A receptor might underlie the observed antidepressant effect. Immobility time in the FST and TST was significantly reduced in animals treated with ethanol extract of Marsilea minuta, and Marsilea minuta (400 mg/kg, p. o.) significantly downregulated the 5-HT2A receptor in the frontal cortex, which is considered a potential antidepressant mechanism (Bhattamisra et al., 2008).

Some natural products can exert antidepressant effects by merely activating dopamine receptors. Carvacrol, given daily for seven consecutive days (12.5 mg/kg p. o.), has been shown to increase levels of dopamine and serotonin in the prefrontal cortex and hippocampus, which may operate via action on dopamine D1 and D2 receptors (Zotti et al., 2013). Through activation of dopamine D1 and D2 receptors, pretreatment of mice with ursolic acid (0.1 mg/kg, p. o.) was able to prevent the antidepressant-like effect (Colla et al., 2014).

Indeed, some natural products exert antidepressant effects by more than one mechanism. Most of them function via multiple mechanisms. Li et al. found that naringenin (10, 20, and 50 mg/kg) possessed antidepressant-like activity in the tail suspension test by elevating NA, 5-HT, and GR levels in the hippocampal region (Yi et al., 2010). The antidepressant-like effect of nobiletin has been proven by Li et al., who found that nobiletin (25, 50, and 100 mg/kg, p. o.) decreased the immobility time in both the FST and TST without locomotor alterations in the OFT, which seems to be mediated by an interaction with the serotonergic (5-HT1A and 5-HT2 receptors), noradrenergic (α1-adrenoceptor) and dopaminergic (D1 and D2 receptors) systems (Yi et al., 2011). Through interaction with the 5-HT2 receptor and α1-and α2-adrenoceptors, amentoflavone significantly (*p* < 0.001) reduced the duration of immobility in the FST and TST, with peak effects observed at 100 and 50 mg/kg, respectively, in comparison to control treatment (Ishola et al., 2012). With the involvement of the serotonergic and noradrenergic and/or dopaminergic systems, rutin (0.three to three mg/kg, p. o.) reduced the immobility time in the TST (Machado et al., 2008). In addition, liquiritin and isoliquiritin, berberine, Hedyosmum brasiliense, podoandin, genipin, bacopaside I, Ptychopetalum olacoides ethanol extract (POEE), punarnavine, *Ceratonia siliqua*, total ethanolic extract of *Convolvulus pluricaulis*, and *Terminalia* bellirica Roxb. fruits have been proven to function via multiple mechanisms, including but not limited to neurotransmitter mechanisms (Irie et al., 2004; Dhingra and Valecha, 2007a; Dhingra and Valecha, 2007b; Peng et al., 2007; Kulkarni and Dhir, 2008; Piato et al., 2009; Agrawal et al., 2011; Zhang et al., 2011; Gonçalves et al., 2012; Liu et al., 2013; Dhingra and Valecha, 2014a; Wang et al., 2014).

Certainly, in addition to the anti-depressant natural products mentioned above, there are several other natural products that exert antidepressant effects by interacting with neurotransmitter systems. The main natural products with this property are shown in Table 1.

**TABLE 1:** Potential natural products for the treatment of PSD by modulating neurotransmitters.

Studied Drugs	Mechanisms	Description of Study along with Doses	Studied Species	Behavioral Tests	Animal Models	References
Ethanol extract of Paeonia lactiflora (EPL)	Mediated via the central monoaminergic neurotransmitter system	Intragastric administration of EPL significantly reduced the duration of immobility in both FST and TST	Male Kunming (KM) mic	FST, TST and OFT	Acute stress model	Mao et al., 2008 (Mao et al., 2008b)
Baicalin	Through MAO A and B inhibition	Baicalin reduced immobility time in TST and FST in mice; Baicalin also decreased immobility time FST in rats	Male wistar rats and male kunming mice	FST and TST	CUMS model	Zhu et al., 2006 (Zhu et al., 2006)
Kaempferol	Inhibitory activity on Monoamine oxidase	Reduces the immobility time in the FST and TST	Sprague-dawley (SD) rats	FST and TST	Acute stress model	Sloley et al., 2000 (Sloley et al., 2000)
Methanol extract of the roots of *Sophora* flavescens	Inhibitory effect on monoamine oxidase (MAO)	The methanol extract of the roots of *Sophora* flavescens showed an inhibitory effect on mouse brain monoamine oxidase (MAO)	ICR male mice	Not application (NA)	NA	Hwang et al., 2005 (Hwang et al., 2005)
Flavonoid naringenin	Elevating NA, 5-HT, and GR levels in the hippocampus region	Naringenin (10, 20, and 50 mg/kg) possessed antidepressant like activity in the tail suspension test	Male ICR mice	FST, TST, and OFT	Acute stress model	Yi et al., 2009 (Yi et al., 2010)
Nobiletin	Seems to be mediated by an interaction with the serotonergic (5-HT1A and 5-HT2 receptors), noradrenergic (α1- adrenoceptor) and dopaminergic (D1 and D2 receptors) systems	Nobiletin decreased the immobility time in both the FST and TST without locomotor alterations in the open-field test (OFT)	Male ICR mice	FST, TST, and OFT	Acute stress model	Yi et al., 2011 (Yi et al., 2011)
Amentoflavone	Interaction with 5-HT2 receptor and α1-, and α2-adrenoceptors	Amentoflavone significantly reduced the immobility time in FST and TST	Swiss albino mice	FST and TST	Acute stress model	Ishola et al., 2012 (Ishola et al., 2012)
Hesperidin	Interplay with the 5-HT (1A) receptors	Hesperidin decreased the immobility time in the FST and TST without affecting the locomotor activity in the open-field test	Male adult swiss mice	FST, TST, and OFT	Acute stress model	Souza et al., 2013 (Souza et al., 2013)
Curcumin	Increased serotonin (5-hydroxytryptamine, 5-HT) as well as dopamine levels (at higher doses), and inhibited the monoamine oxidase enzymes (both MAO-A and MAO-B, higher doses)	Curcumin was active in mouse FST and TST	Male Laca mice	FST	Acute stress model	Kulkarni et al., 2008 (Kulkarni et al., 2008)
Fisetin	Inhibition of MAO-A	Fisetin inhibited the immobility time in both FST and TST	Male ICR mice	FST and TST	Acute stress model	Zhen et al., 2012 (Zhen et al., 2012)
Rutin	Involvement of the serotonergic and noradrenergic and/or dopaminergic systems	The administration of rutin reduced the immobility time in the TST	Male swiss mice	FST, TST and OFT	Acute stress model	Machado et al., 2008 (Machado et al., 2008)
Ferulic acid	Via inhibiting serotonin, norepinephrine and dopamine reuptakes, regulating HPA axis and increasing ghrelin	Ferulic acid achieve anti-depressant effect through acting the Serotoninergic pathway	Male sprague–daw	FST and OFT	Acute stress model	Zhang et al., 2011 (Zhang et al., 2011)
Trans-resveratrol	Related to serotonergic and noradrenergic activation, inhibition of MAO-A	Trans-Resveratrol significantly decreased the immobility time in mouse models of despair tests	Male ICR mice	FST and TST	Acute stress model	Xu et al., 2010 (Xu et al., 2010)
Liquiritin and isoliquiritin	Increase 5-HT and NE in the mouse hippocampus, hypothalamus and cortex	Both liquiritin and isoliquiritin significantly reduced the immobility time in the FST and TST in mice	Mice	FST and TST	Acute stress model	Wang et al., 2008 (Wang et al., 2008b)
Piperine and antiepilepsirine	Elevated the dopamine level in striatum, hypothalamus and hippocampus; increased the serotonin level in the hypothalamus and hippocampus; a minor MAO inhibitory activity	After 2 weeks of chronic administration, PIP and AES significantly reduced the duration of immobility in both FST and TST, without accompanying changes in locomotor activity in the open-field test	Male ICR mice	FST and TST	Acute stress model	Li et al., 2007 (Li et al., 2007)
Berberine	Related to the increase in NA and 5-HT levels in the hippocampus and frontal cortex	The results show that BER significantly reduced the immobility time in the FST and TST	Male ICR mice	FST and TST	Acute stress model	Peng et al., 2007 (Peng et al., 2007)
Neferine	Acting on HT1a receptor	Elicited anti-immobility effects in mice	Male ICR mice	FST	Acute stress model	Sugimoto et al., 2010 (Sugimoto et al., 2010)
Palmatine	A decrease in MAO-A activity	Palmatine significantly decreased immobility periods of unstressed and stressed mice in the FST and TST	Male swiss albino	FST and TST	CUMS model	Dhingra et al., 2014 (Dhingra and Bhankher, 2014)
Punarnavine	Decreased monoamine oxidase (MAO-A) activity; decrease in plasma corticosterone levels	It decreases immobility periods in the FST	Mice	FST	CUMS model	Dhingra et al., 2014 (Dhingra and Valecha, 2014b)
Hedyosmum brasiliense and pod	Dependent on the serotonergic, noradrenergic and dopaminergic systems	H. brasiliense and podoandin decreased the immobility time in the FST, without any accompanying changes in ambulation in the open-field test	Male swiss mice	FST and OFT	Acute stress model	Genclaves et al., 2012 (Gonçalves et al., 2012)
Carvacrol	Action on dopamine D1 and D2 re	Carvacrol, administered for seven consecutive days, was able to increase dopamine and serotonin levels in the prefrontal cortex and hippocampus	Adult male wistar r	FST	Acute stress model	Zotti et al., 2013 (Zotti et al., 2013)
Genipin	Elevates 5-HT and NE level	Pre-treatments with genipin significantly increased the levels of 5-HT, NE and decreased the level of 5-HIAA in the hippocampus	Male sprague dawl	OFT	CUMS model	Wang et al., 2014 (Wang et al., 2014)
Ursolic acid	Activation of dopamine D1 and D2 receptors	Pre-treatment of mice with UA was able to prevent the antidepressant-like effect	Swiss mice	TST and OFT	Acute stress model	Colla et al., 2014 (Colla et al., 2014)
β- amyrin palmitate	Activate noradrenergic activity	Reduction in immobility time of FST and TST model	Male ddy strain mice	FST	Acute stress model	Subarnas et al., 1993 (Subarnas et al., 1993)
Bacopaside I	Might be related to both antioxidant activation and noradrenergic activation	Bacopaside I significantly decreased the immobility time in mouse models of despair tests, but it did not influence locomotor activity	Male ICR mice	FST and TST	Acute stress model	Liu et al., 2013 (Liu et al., 2013)
l-theanine (γ-glutamylethylamide)	Might be mediated by interaction with the central monoaminergic system	l‐theanine significantly reduced the immobility time in both the FST and TST	Male kunming mice	FST, TST and OFT	Acute stress model	Yin et al., 2011 (Yin et al., 2011)
Ptychopetalum olacoides ethanol extract (POEE)	Possibly mediated by β-adrenergic and D1 dopamine receptors	POEE resulted in a significant and dose-related anti-immobility effect in both FST and TST	CF1 mice	FST and TST	Acute stress model	Piato et al., 2009 (Piato et al., 2009)
Acanthopanax senticosus extract	May be mediated via the central monoaminergic neurotransmitter system and CREB protein expression	Intragastric administration of ASE significantly reduced the duration of immobility in both FST and TST	Male kunming mic	FST, TST, and OFT	Acute stress model	Jin et al., 2013 (Jin et al., 2013)
Laetispicine	Possibly act on the CNS monoaminergic neurotransmitters	A significant and dose-dependent decrease in the immobility time, as evaluated by the FST, was observed after laetispicine administration, suggesting an antidepressant effect	KM mice	FST and OFT	Acute stress model	Yao et al., 2009 (Yao et al., 2009)
*Hypericum* caprifoliatum	Monoamine uptake inhibition	The antidepressant-like effect of H. caprifoliatum on the FST is due to an increase in monoaminergic transmission, resulting from monoamine uptake inhibition	Adult male wistar rats	FST	Acute stress model	Viana et al., 2005 (Viana et al., 2005)
Hydroalcoholic extract of Rosmarinus officinalis	Mediated by an interaction with the monoaminergic system	The extract of R. officinalis produced an antidepressant-like effect, since the acute treatment of mice with the extract by p.o. route significantly reduced the immobility time in the FST and TST	Male swiss mice	FST, TST, and OFT	Acute stress model	Machado et al., 2009 (Machado et al., 2009)
Ethanolic extract from Tabebuia avellanedae	Involvement of the monoaminergic system	The extract from T. avellanedae produced an antidepressant-like effect, in the FST and in the TST	Adult female swiss mice	FST, TST, and OFT	Acute stress model	Freitas et al., 2009 (Freitas et al., 2010)
Methanolic extract from Bupleurum falcatum	Involves the serotonergic and noradrenergic systems	the methanolic extract from Bupleurum falcatum significantly reduced the total duration of immobility in the TST, while individual differences in locomotor activities between experimental groups were not observed in the OFT	Male BALB/c mice	TST and OFT	Acute stress model	Kwon et al., 2010 (Kwon et al., 2010)
Schinus molle	Involvement of the monoaminergi	The immobility time in the TST was significantly reduced by the extract of Schinus mole, without accompanying changes in ambulation when assessed in an open-field test	Male swiss mice	TST	Acute stress model	Machado et al., 2007 (Machado et al., 2007)
Ascorbic acid (vitamin C)	Interaction with the monoaminergi	Ascorbic acid produced an antidepressant-like effect in the TST, but not in the FST, without altering the locomotor activity	Adult swiss mice	FST, TST, and OFT	Acute stress model	Binfaré et al., 2009 (Binfaré et al., 2009)
Scopoletin	Involvement of monoaminergic sy	Scopoletin reduced the immobility time in the TST, but not in the FST	Female swiss mice	FST, TST, and OFT	Acute stress model	*Capra* et al., 2010 (Capra et al., 2010)
Ebselen	Involvement of the monoaminergic system	Ebselen decreased the immobility time in the FST without accompanying changes in ambulation in the open-field test. In contrast, the administration of ebselen did not produce any effect in the TST	Adults male swiss mice	FST, TST, and OFT	Acute stress model	Posser et al., 2009 (Posser et al., 2009)
Hyperfoliatin	Monoamine uptake inhibition	In the FST, hyperfoliatin dose-dependently reduced immobility time	Male swiss albinos CD1 mice	FST	Acute stress model	Rego et al., 2007 (do Rego et al., 2007)
1-(m-Chlorophenyl) piperazine	Stimulating the neuronal 5-HT2A	1-(m-Chlorophenyl) piperazine exhibited depressant-like effects in FST and TST (in mice), without influencing the locomotor status	Male swiss albino mice	FST and TST	Acute stress model	Rajkumar et al., 2009 (Rajkumar et al., 2009)
Total glycosides of peony (TGP)	Inhibited the activities of monoam	Intragastric administration of TGP caused a significant reduc of immobility time in both FST and TST	Male ICR mice	FST, TST, and OFT	Acute stress model	Mao et al., 2008 (Mao et al., 2008a)
*Hypericum* canariense L	Direct or indirect facilitation of the central serotonergic transmission	The butanol and chloroform fractions from *Hypericum canariense* and *Hypericum glandulosum* possess antidepressant-like effects in mice	Albino swiss mice	FST	Acute stress model	Mateo et al., 2005 (Sánchez-Mateo et al., 2005)
*Tagetes* lucida (Asteraceae)	The involvement of serotonergic br	The extract of *Tagetes* lucida significantly reduced immobility and increased swimming without affecting climbing behavior in the FST	Male wistar rats	FST and OFT	Acute stress model	Cruz et al., 2008 (Guadarrama-Cruz et al., 2008)
Marsilea minuta Linn	Down regulated 5-HT2A receptor	Immobility time in FST and TST was significantly reduced by ethanol extract of Marsilea minuta treated animals and Marsilea minuta significantly down regulated 5-HT2A receptor in frontal cortex	Swiss albino mice	FST and TST	LH model	Bhattamisra et al., 2008 (Bhattamisra et al., 2008)
Isorhynchophylline (IRN)	Inhibition of monoamine oxidases	Intragastric administration of IRN caused a significant reduction of immobility time in both FST and TST, while IRN did not stimulate locomotor activity in the open-field test	Male BALB/c mice	FST, TST, and OFT	Acute stress model	Xian et al., 2017 (Xian et al., 2017)
Punarnavine	Interaction with monoaminergic and GABAergic systems	Antidepressant-like effect of the extract of punarnavine were found to be comparable to fluoxetine	Swiss albino mice	FST and TST	Acute stress model	Dhingra et al., 2014 (Dhingra and Valecha, 2014a)
Ceratonia siliqua L. (Fabaceae)	Mediated by dopamine and noradrenaline	The immobility time in the TST and FST were significantly reduced by CS	Male albino mice	FST and TST	Acute stress model	Agrawal et al., 2011 (Agrawal et al., 2011)
The chloroform fraction of the total ethanolic extract of *Convolvulus* pluricaulis	Interaction with the adrenergic, dopaminergic, and serotonergic systems	The chloroform fraction of the total ethanolic extract of *Convolvulus* pluricaulis significantly reduced the immobility time in both FST and TST	Swiss male albino	FST and TST	Acute stress model	Dhingra et al., 2007 (Dhingra and Valecha, 2007a)
*Terminalia* bellirica Roxb. fruits	Interaction with adrenergic, dopaminergic and serotonergic systems	Aqueous extract in a dose-dependent manner and ethanolic extract significantly reduced the immobility time of mice in both FST and TST	Mice	FST and TST	Acute stress model	Dhingra et al., 2007 (Dhingra and Valecha, 2007b)
Cocoa polyphenolic extract	Elevate antioxidative enzyme activities and uptake of brain monoamine neurotransmitters	Cocoa polyphenolic extract significantly reduced the duration of immobility	Male wistar–unilev	FST and OFT	Acute stress model	Messaoudi et al., 2008 (Messaoudi et al., 2008)

### 4.2 Anti-inflammation Therapy

Although much convincing evidence has established that inflammatory responses, as important biological events, have a strong relationship with depression, few existing studies have found the exact anti-inflammatory effects of natural products in the treatment of PSD. In a study of an olfactory bulbectomy (OBX) rat model, researchers found that after a surgical recovery period of 2 weeks, treatment with quercetin (40, 80 mg/kg; p. o., 14 days) significantly prevented OBX-induced behavioral, biochemical, molecular, and histopathological alterations associated with suppression of the oxidative-nitrosative stress-mediated neuroinflammation-apoptotic cascade (Rinwa and Kumar, 2013). The main natural products are shown in Table 2.

**TABLE 2:** Potential natural products for the treatment of PSD by anti-inflammation and modulating HPA axis.

Studied Drugs	Mechanisms	Description of Study along with Doses	Studied Species	Behavioral Tests	Animal Models	References
Quercetin	Anti-inflammatory	After a surgical recovery period of 2 weeks, treatment with quercetin significantly prevented OBX-induced behavioral, biochemical, molecular and histopathological alterations	Adult male wistar rats	FST	OBX model	Rinwa et al., 2013 (Rinwa and Kumar, 2013)
Icariin	Improving the abnormalities in the HPA axis functions	Enhances antioxidant and anti-inflammatory activity	Male wistar rats	Sucrose preference test (SPT)	CUMS model	Pan et al., 2007 (Pan et al., 2007)
Paeoniflorin	The modulation of the HPA axis and up-regulation of serotonergic and noradrenergic systems	Paeoniflorin treatment markedly increased sucrose consumption and decreased serum corticosterone and adrenocorticotropic hormone levels in the CUS-treated rats	Male SD rats	SPT	CUMS model	Qiu et al., 2013 (Qiu et al., 2013)
Mitragynine	Acting on HPA axis	Mitragynine significantly reduced the immobility time of mice in both FST and TST without any significant effect on locomotor activity in OFT	Not application	FST, TST, and OFT	Acute stress model	Ldayu et al., 2011 (Idayu et al., 2011)
Total glycosides of peony	Mediated by modulating the functional status of HPA axis and increasing the expression of BDNF in brain tissues	Daily intragastric administration of total glycosides during the 6 weeks of CUMS significantly suppressed behavioral and biochemical changes induced by CUMS	Male ICR mice	FST	CUMS model	Mao et al., 2009 (Mao et al., 2009)
Ethanolic extract from Curcuma longa	Regulations of neurochemical and neuroendocrine systems, such as monoamine neurotransmitter levels, the HPA axis action	The ethanolic extract was found to reduce the duration of immobility in the mouse FST	Male ICR strain of mice	FST and OFT	Acute stress model	Xia et al., 2007 (Xia et al., 2007)
Aqueous extract of Camellia euphlebia (AEC)	Via modulation of the hypothalamic-pituitary-adrenal axis and brain monoaminergic systems	Mice administered AEC showed significantly reduced immobility duration in FST and TST, whilst exhibiting no apparent changes in locomotor activity	Kunming (KM) mice and sprague-dawley (SD) rats	FST and TST	CUMS model	He et al., 2018 (He et al., 2018)

### 4.3 Therapeutic Strategy of Modulation of the HPA Axis

HPA axis activation is quite common after stroke and leads to elevated glucocorticoid levels. Several types of research proved that some natural products create anti-depressant activity via the modulation of the HPA axis, to speak exactly, downregulating the level of HPA. Pan et al. found that icariin, a major constituent of flavonoids isolated from Epimedium brevicornum, possessed potent anti-inflammatory activity at a dose of 20 or 40 mg/kg, which was in part mediated by improving abnormalities in HPA axis functions (Pan et al., 2007). Acting on the HPA axis, mitragynine at a dose of 10 mg/kg and 30 mg/kg i. p. injected extremely reduced the immobility time of mice in both the FST and TST and significantly reduced the release of corticosterone (Idayu et al., 2011). In addition, paeoniflorin, total glycosides of peony, ethanolic extract from Curcuma longa, and aqueous extract of Camellia euphlebia (AEC), which act via multiple mechanisms, have also been investigated previously (Xia et al., 2007; Mao et al., 2009; Qiu et al., 2013; He et al., 2018). The main natural products are shown in Table 2.

### 4.4 Therapeutic Strategy for Modulating Central Neuroplasticity

Previous studies proved that an impairment in nerve growth is largely responsible for depression, and the impairment of the brain’s ability to promote neurogenesis underlies depression (Yan et al., 2016). According to this hypothesis, new neuronal connections in the hippocampus contribute to mood regulation and the pharmaceutical effect of antidepressants. BDNF, as an endogenous neurotrophic factor, is necessary to modulate neuronal plasticity. Several antidepressant effects of natural products correlate with increased BDNF synthesis and activity in the hippocampus.

In previous studies, Walker et al. found that the PI3K/Akt/mTOR pathway plays a critical role in neuroprotection (Walker et al., 2019). On this basis, Zhang et al. further demonstrated that Sinisan (SNS) protects neurons from corticosterone-induced injury by inhibiting autophagy through induction of the PI3K/AKT/mTOR pathway (Zhang et al., 2021b). In addition, other natural products produce an anti-depressant effect by inducing BDNF, which further promotes neurogenesis. Lee et al. proved the potential for Angelica gigas extract (AGN) to effectively treat repeated injection of corticosterone (CORT)-related depression and anxiety-like symptoms, possibly by modulating the central noradrenergic system and regulating the expression of BDNF (Lee et al., 2015). Zhang et al. found that ethanol extract of Gardenia jasminoides exerts rapid antidepressant effects that are associated with a rapid increase in BDNF expression in the hippocampus (Zhang et al., 2015b). Additionally, hyperoside, baicalein, albiflorin, tetrandrine, cannabidiol, hyperforin, protopanaxadiol (PPD), ginsenoside Rg1, and eugenol have been proven to produce antidepressant effects by elevating the expression of BDNF (Irie et al., 2004; Zanelati et al., 2010; Xiong et al., 2011; Jiang et al., 2012; Zheng et al., 2012; Gao et al., 2013; Gibon et al., 2013; Wang et al., 2016; Jiang et al., 2019; Silote et al., 2019). The main natural products in this section are shown in Table 3.

**TABLE 3:** Potential natural products for the treatment of PSD by modulating the central neuroplasticity.

**Studied Drugs**	**Mechanisms**	**Description of Study along with Doses**	**Studied Species**	**Behavioral Tests**	**Animal Models**	**References**
Sinisan	Activation of PI3K/AKT/mTOR pathway	SNS protects neurons against corticosterone-induced injury by inhibiting autophagy through induction of PI3K/AKT/mTOR pathway	Sprague-dawley (SD) rats	NA	NA	Zhang et al., 2021 (Zhang et al., 2021b)
Angelica gigas extract	Possibly by modulating the central noradrenergic system and regulation of BDNF expression	Indicate the potential for Angelica gigas extract (AGN) to effectively treat Repeated injection of corticosterone (CORT)-related depression and anxiety-like symptoms, possibly via modulation of the central noradrenergic system and regulation of BDNF expression	Sprague-dawley (SD) rats	FST and OFT	Acute stress model	Lee et al., 2015 (Lee et al., 2015)
Ethanol Extract of Gardenia jasminoides	Via instant enhancement of brain-derived neurotrophic factor (BDNF) expression in the hippocampus	Ethanol Extract of Gardenia jasminoides has rapid antidepressant effects, which are associated with acutely increased expression of BDNF in the hippocampus	Kunming mice	TST and OFT	LH model	Zhang et al., 2015 (Zhang et al., 2015b)
Astilbin (AST)	Up-regulation of monoaminergic neurotransmitters (5-HT and DA) and activation of the BDNF signaling pathway	Chronic administration of AST reduced depressive-like behaviors of mice without affecting locomotor activity	Adult male C57BL/6 J mice	FST, TST, and OFT	CUMS model	LV et al., 2014 (Lv et al., 2014)
Hyperoside	Elevation the expression of BDNF and CREB through the signal pathway AC–cAMP–CREB.	Hyperoside attenuated the intracellular Ca2+ overloading in PC12 cells induced by corticosterone	Vitro model of PC12 cells	NA	NA	Zheng et al., 2012 (Zheng et al., 2012)
Baicalein	Stimulates the levels of brain-derived neurotrophic factor (BDNF) expression	Acute application of Bai significantly reduced the immobility time in the FST and TST of mice	Adult male kunming (KM) mice	FST, TST, and OFT	CUMS model	Xiong et al., 2011 (Xiong et al., 2011)
Albiflorin	Closely related to the hippocampal 5-HT/NE increase and BDNF expression	7 days treatment with albiflorin significantly decreased immobility time in FST and TST without alter the locomotor activity in mice	Male ICR mice	FST, TST, and OFT	CUMS model	Wang et al., 2016 (Wang et al., 2016)
Tetrandrine	Regulation of the central monoaminergic neurotransmitter system and the levels of BDNF.	Reduces immobility time in both the FST and TST	Male ICR mice	FST, TST, and OFT	CUMS model	Gao et al., 2013 (Gao et al., 2013)
Cannabidiol	Increase brain-derived neurotrophic factor (BDNF) levels	Cannabidiol induces dose-dependent antidepressant-like effects	Male swiss mice	FST	Acute stress model	Zanelati et al., 2010 (Zanelati et al., 2010)
Ginsenoside Rg1	Activation of the BDNF signalling pathway and up-regulation of hippocampal neurogenesis	Ginsenoside Rg1 exhibited antidepressant-like activity in the FST and TST in mice without affecting locomotor activity	Adult male C57BL/6 J mice	FST, TST, and OFT	CUMS model	Jiang et al., 2012 (Jiang et al., 2012)
Hyperforin	Acts on the cortical BDNF/TrkB pathway	Enhancing BDNF expression in the frontal cortex	Cortical neurons and male C57Bl6/J mice	NA	NA	Gibon et al., 2013 (Gibon et al., 2013)
Protopanaxadiol	Enhance the PI3 K/Akt/mTOR‐mediated BDNF/TrkB pathway	Protopanaxadiol (PPD) exerts antidepressant‐like effects in mice with CSDS‐ induced depression	Adult male C57BL/6 J and CD1 mice	FST and TST	CSDS model	Jiang et al., 2019 (Jiang et al., 2019)
Eugenol	Induce brain-derived neurotrophic factor (BDNF)	Eugenol has an antidepressant-like activity comparable to that of imipramine in the FST and TST	Male ddY mice	FST and TST	Acute stress model	Irie et al., 2004 (Irie et al., 2004)

### 4.5 Therapeutic Strategies Targeting Other Mechanisms

Apart from the main mechanisms described above, natural products can also produce anti-depressant effects via other mechanisms. Some studies did not find an exact mechanism for natural products in anti-depressant procession. Through an inverse-agonistic mechanism located in benzodiazepine receptors, the treatment of animals with harmane (5–15 mg/kg, i. p.), norharmane (2.5–10 mg/kg, i. p.), and harmine (5–15 mg/kg, i. p.) reduced the immobility time in a dose-dependent manner (Farzin and Mansouri, 2006). Chen et al. found that a single bilateral intra-ventrolateral orbital cortex (VLO) infusion of sanguinarine (2.5, 5, or 10 g/0.5 L per side) significantly reduced immobility time in the FST in a dose-dependent fashion, which may act through a decrease in the expression of Mkp-1 and an increase in ERK activation (Chen et al., 2012). By decreasing the expression of Mkp-1 and increasing ERK activation, a single bilateral intra-ventrolateral orbital cortex (VLO) infusion of sanguinarine (2.5, 5, or 10 g/0.5 L per side) significantly reduced immobility time in the FST in a dose-dependent fashion (Chen et al., 2012). Action on cannabinoid receptor subtype 2, β-caryophyllene, at doses of 25, 50, and 100 mg/kg mitigates stress-related changes in the hippocampal region (Hwang et al., 2020). Mediated by both the k-opioid and endocannabinoid systems, salvinorin A, given s. c. (0.001–1,000 mg·kg-1), exhibited both anxiolytic- and antidepressant-like effects (Braida et al., 2009). Blocking the activities of high mobility group box 1 (HMGB1) may be one of the principal ways in association with the antidepressant effect of glycyrrhizin (Wang et al., 2018). The aqueous root extract of Securidaca longipedunculata (Polygalaceae) produced a significant naloxone-reversible antidepressant-like effect in the FST, with possible involvement of opioidergic pathways (Adebiyi et al., 2006).

In addition, guarana, hydroethanolic of extract of Aloysia polystachya (CEAp), Lafoensia pacari A. St.-Hil. (Lythraceae), D-004, asiaticoside, hydroalcoholic extract of Gastrodia elata, hydroethanolic and dichloromethanic extracts of *Sonchus oleraceus*, and ethanolic extract of *Hypericum perforatum* produce anti-depressant effects with no clear mechanism (Kumar et al., 2000; Bach-Rojecky et al., 2004; Campos et al., 2005; Zhou et al., 2006; Hellión-Ibarrola et al., 2008; Liang et al., 2008; Carbajal et al., 2009; Galdino et al., 2009; Vilela et al., 2010). The main natural products in this section are shown in Table 4.

**TABLE 4:** Potential natural products for the treatment of PSD by other mechanisms.

Studied Drugs	Mechanisms	Description of Study along with Doses	Studied Species	Behavioral Tests	Animal Models	References
Harmane, norharmane and harmine	Inverse-agonistic mechanism located in the benzodiazepine receptors	Treatment of animals with harmane, norharmane and harmine reduced dose-dependently the time of immobility	Male swiss-webst	FST	Acute stress model	Farzin et al., 2006 (Farzin and Mansouri, 2006)
Sanguinarine	A decrease in expression of Mkp-1 and increase in ERK activation	A single bilateral intra--the ventrolateral orbital cortex (VLO) infusion of Sanguinarine significantly reduced immobility time in the FST in dose-dependent fashion	Male sprague-da	FST	Acute stress model	Chen et al., 2012 (Chen et al., 2012)
β-caryophyllene---β	Action on cannabinoid receptor subtype 2	Mitigates the stress-related changes in the hippo-campus region	Male swiss mice	TST and FST	Acute stress model	Hwang et al., 2020 (Hwang et al., 2020)
Salvinorin A	Mediated by both k-opioid and endocannabinoid systems	Salvinorin A exhibited both anxiolytic- and antidepressant-like effects	Adult male sprague-dawley rats and swiss mice	TST and FST	Acute stress model	Braida et al., 2009 (Braida et al., 2009)
Glycyrrhizin	Blocking the activities of HMGB1	Reduce immobility time of mice in FST and TST model	BABL/c mice	FST and TST	CUMS model	Wang et al., 2018 (Wang et al., 2018)
Guarana	Nuclear	Guarana significantly reduced the duration of immobility in the FST suggesting an antidepressant-like effect in mice	Mice	FST	Acute stress model	Campos et al., 2005 (Campos et al., 2005)
Hydro-ethanolic of extract Aloysia polystachya (CEAp)	Unclear	A single dose of hydro-ethanolic of extract Aloysia polystachya (CEAp) provoked a significant reduction of the immobility time of male mice in the FST	Swiss albino male	FST	Acute stress model	Lbarrola et al., 2008 (Hellión-Ibarrola et al., 2008)
Lafoensia pacari A. St.-Hil. (Lythraceae)	Unclear	The daily treatment for 21 days with ethanolic extract of Lafoensia pacari (PEtExt) increased the latency to immobility and decreased the immobility time, PEtExt 0.1 only decrease the immobility time	Male swiss mice	FST, TST, and OFT	Acute stress model	Galdino et al., 2009 (Galdino et al., 2009)
Aqueous root extract of Securidaca longepedunculata (polygalaceae)	With possible involvement of opioidergic pathways	The extract also produced a significant (*p* < 0.05) naloxone reversible antidepressant like effect in the FST	Swiss albino mice	FST	Acute stress model	Adebiyi et al., 2006 (Adebiyi et al., 2006)
D-004	Unclear	D-004 administered orally for 30 days reduced the immobility in the FST and the TST in mice, and had no effect on other behavioural tests in mice	Adult swiss OF1	FST and TST	Acute stress model	Garbajal et al., 2009 (Carbajal et al., 2009)
Asiaticoside	Unclear	Asiaticoside significantly decreased immobility time in FST and TST, but its mechanism is still unclear and required to be further investigated (Dhingra and Valecha, 2014a)	Male swiss mice	FST, TST, and OFT	CUMS model	Liang et al., 2008 (Liang et al., 2008)
Hydroalcoholic extract of Gastrodia elata	Unclear	G. elata aqueous ethanol extract significantly reduced the immobility duration in FST and TST	Male kunming mi	FST, TST and OFT	Acute stress model	Zhou et al., 2006 (Zhou et al., 2006)
Hydroethanolic and dichloromethanic extracts *Sonchus* oleraceus	Unclear	Hydroethanolic and dichloromethanic extracts *Sonchus* oleraceus reduced the immobility duration in FST and TST, while the mechanism has not been known	Adult male swiss mice	FST and TST	Acute stress model	Vilela et al., 2010 (Vilela et al., 2010)
Ethanolic extract of *Hypericum perforatum*	Unclear	*H. perforatum* extract displays dose-dependent antidepressant effect	Mice	OFT	Acute stress model	Kumar et al., 2000 (Kumar et al., 2000)

## 5 Conclusion and Perspectives

PSD is a major disease affecting patients’ life quality (Bai and Wang, 2019; Sutoko et al., 2020). It is also a critical problem for patients because PSD increases suicide and mortality (Mitchell et al., 2017; Tang et al., 2020). For the last few decades, antidepressants such as monoamine oxidase inhibitors, tricyclic antidepressants, and selective serotonin reuptake inhibitors have been used clinically (Li et al., 2018). However, most of these antidepressants have many serious adverse side effects (Sarko, 2000; Ferguson, 2001; Coupland et al., 2011). Therefore, many alternative therapeutic strategies for the treatment of depression have been reported. In particular, many studies have reported that natural products with antioxidant and anti-inflammatory effects could improve neurodegeneration. In recent years, strong evidence from different scientific studies has supported the idea that natural products may be new therapeutic tools against depression due to more advantages than synthetic prescription drugs.

The literature review summarizes the current literature on treatments with natural products for PSD. And the purpose of this review is to prospect the potential of natural products in the treatment of PSD and make more specific requests for subsequent research. However, our literature review is not without limitations. Firstly, the mechanisms of some of the original studies we included are merely at the hypothesis stage and are not very clear. Secondly, tremendous studies are still in the experimental stage, and there is still a lack of clinical studies to assess the safety and potency of phytochemicals with prospective antidepressant activities. Thirdly, we have emphasized the adverse effects of traditional treatment for PSD, however, we did not address any potential side effects of the reported natural compounds. Being “natural” does not necessarily mean that they are free of toxicity or side effects. In future studies, a large number of studies that confirm the safety and effectiveness of natural products in the treatment of PSD should be designed. Those natural products that have been proved safe and effective may be used in clinical applications widely.
